# Infection of human induced pluripotent stem cells by an oncogenic herpesvirus

**DOI:** 10.3389/fcimb.2025.1563440

**Published:** 2025-03-13

**Authors:** Jiaojiao Fan, Zhen Lin, Huiliang Zhang, Lu Dai, Zhiqiang Qin

**Affiliations:** ^1^ Department of Pathology, Winthrop P. Rockefeller Cancer Institute, University of Arkansas for Medical Sciences, Little Rock, AR, United States; ^2^ Department of Pathology and Laboratory Medicine, Tulane University Health Sciences Center, Tulane Cancer Center, New Orleans, LA, United States; ^3^ Department of Medical Genetics and Molecular Biochemistry, Lewis Katz School of Medicine, Temple University, Philadelphia, PA, United States

**Keywords:** KSHV, hiPSCs, stem cell, herpesvirus, infection

## Abstract

**Objective:**

As one of the major human oncogenic viruses, Kaposi’s Sarcoma-associated Herpesvirus (KSHV) is closely related to several cancers such as Kaposi’s sarcoma (KS) and primary effusion lymphoma (PEL). KSHV can infect a broad tropism of human primary cells *in vitro* and *in vivo*. Embryonic stem cell-like pluripotent stem cells can be generated by the simultaneous introduction of several factors, into somatic cells, yielding induced pluripotent stem (iPS) cells. However, it remains unclear whether human induced pluripotent stem cells (hiPSCs) are permissive to KSHV and how this oncogenic virus infection may affect cellular gene profile.

**Methods:**

In the current study, we examined whether hiPSCs were permissive to KSHV infection. The flow cytometry was used to assess the impacts of KSHV infection on hiPSCs viability and apoptosis. The Illumina RNA-Sequencing was used to determine cellular gene profile changed in KSHV-infected hiPSCs and lytically induced cells.

**Results:**

We report that KSHV successfully establishes latent infection in hiPSCs, which can be completely induced to lytic reactivation and release infectious virions. KSHV *de novo* infection arrests the growth of hiPSCs through inducing cell apoptosis. Transcriptomic analysis revealed significant changes in global cellular gene expression in KSHV-infected hiPSCs as well as lytically induced cells.

**Conclusion:**

Our findings demonstrate hiPSCs as a powerful tool to explore the potential impacts of KSHV infection on stem cell functions and virus pathogenesis in stem cell differentiated cells.

## 1 Introduction

Kaposi’s Sarcoma-associated Herpesvirus (KSHV) was first discovered in Kaposi’s sarcoma (KS) lesion from an AIDS patient, and this virus serves as the causative agent of KS, primary effusion lymphoma (PEL), and multicentric Castleman disease (MCD) (Mesri et al., 2014). Recent studies have linked KSHV to the KSHV inflammatory cytokine syndrome (KICS) and osteosarcoma (Polizzotto et al., 2012; Chen et al., 2021). Like other herpesviruses, KSHV life cycle comprises two main phases: latency and lytic replication. During latency, only limited viral latent genes and viral microRNAs are expressed. Once being stimulated to the lytic phase, a wide array of viral genes is expressed, culminating in the assembly of virion particles and finally the release of mature progeny viruses to infect new cells (Avey et al., 2015). KSHV infects a variety of *in vivo* target cells, such as endothelial cells, B cells, monocytes, epithelial cells, and keratinocytes. KSHV also infects a diversity of *in vitro* target cells and establishes *in vitro* latency in many of these cell types (Chakraborty et al., 2012). Mesenchymal stem cells (MSCs) exhibit the potential to differentiate into a variety of mesodermal cell lineages, such as adipocytes, chondrocytes, osteocytes, and myocytes (Ankrum et al., 2014). Interestingly, KSHV have been found to infect human primary MSCs derived from diverse organs, including bone marrow, adipose tissue, dental pulp, gingiva tissue, and exfoliated deciduous teeth (Lee et al., 2016). Also, KSHV can efficiently infect, transform, and reprogram rat primary MSCs into KS-like tumor cells (Jones et al., 2012).

Takahashi and Yamanaka first time demonstrated induction of pluripotent stem cells (iPSCs) from mouse embryonic or adult fibroblasts by introducing four factors, Oct3/4, Sox2, c-Myc, and Klf4, under embryonic stem cell culture conditions (Takahashi and Yamanaka, 2006). This groundbreaking discovery offers significant potential in disciplines such as biology, pathophysiology, and cellular regenerative medicine. As a result, iPSCs derived differentiated cells (e.g., dendritic cells and macrophages) have become a pioneering avenue for cell therapy research (Senju et al., 2011; Wu et al., 2024). However, it remains unclear whether human iPSCs (hiPSCs) are permissive to KSHV and how this oncogenic virus infection may affect cellular gene profile. These interesting questions will be addressed in the current study using the *in vitro* culture system, next-generation sequencing (NGS) analysis and different functional assays.

## 2 Materials and methods

### 2.1 Cell culture, reagents and infection protocols

**Figure 1:**
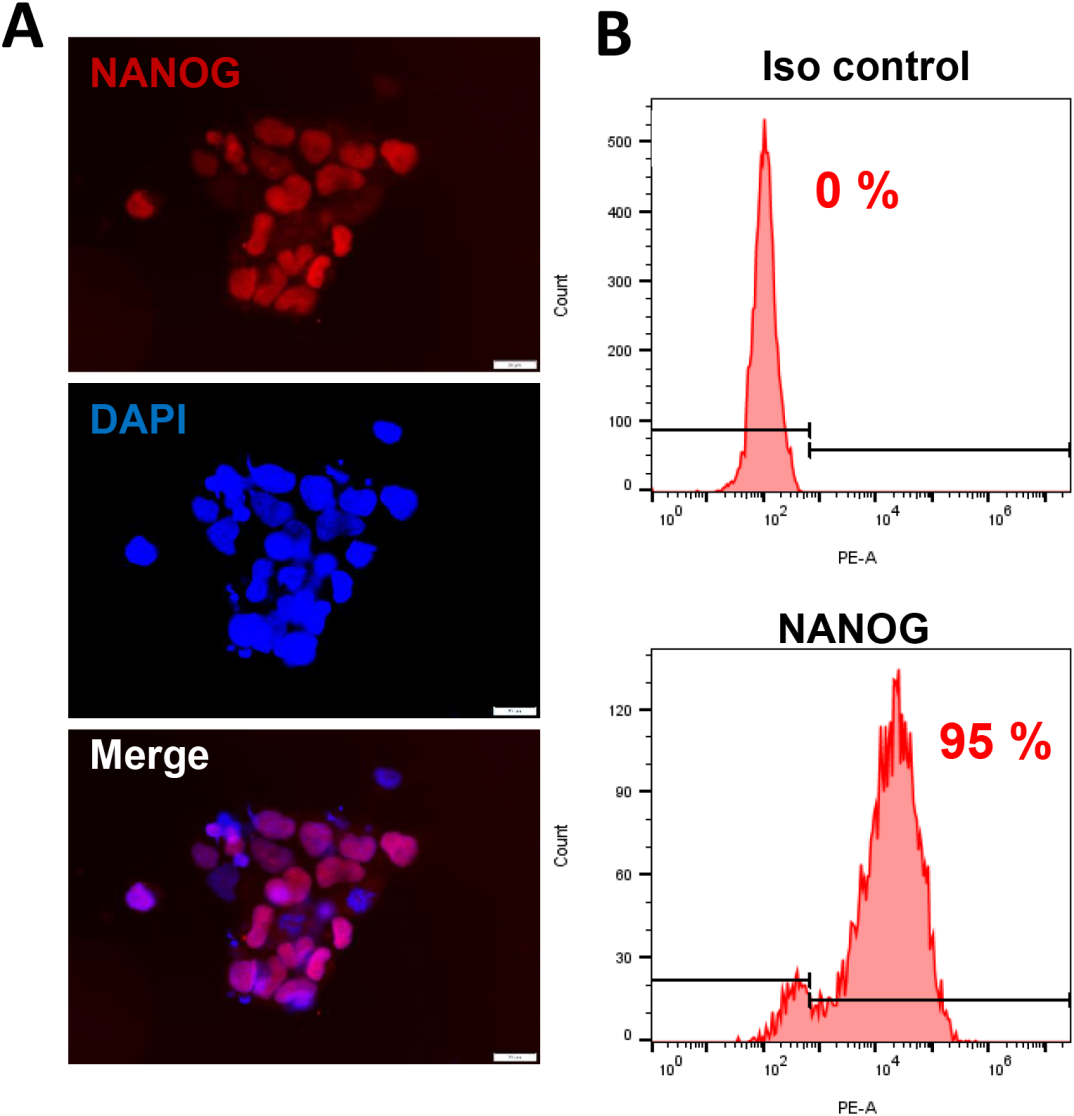
The culture of human induced pluripotent stem cells (hiPSCs). **(A, B)** The hiPSCs were cultured in mTeSR1 medium with supplements (Stemcell Technologies). The expression of stem cell marker, NANOG, was detected by using immunofluorescence and flow cytometry (isotype Ab as a negative control), respectively. Scale bar, 20 μm.

KSHV+ PEL cell line BCBL-1 was kindly provided by Dr. Dean Kedes (University of Virginia) and cultured in RPMI 1640 media (Gibco) with supplemented with 10% fetal bovine serum (FBS), 10 mM HEPES, 100 U/mL penicillin, 100 µg/mL streptomycin, 2 mM L-glutamine, 0.05 mM β-mercaptoethanol, and 0.02% (wt/vol) sodium bicarbonate. For KSHV infection experiments, BCBL-1 cells were incubated with 0.6 mM valproic acid for 4-6 days, then the virions was purified from the culture supernatant by using the ultracentrifugation at 20,000 × g for 3 h, 4°C. The viral pellets were resuspended in 1/100 of the original volume with cell culture medium. The infectious titers of virus were determined as described previously (Qin et al., 2010). Human iSLK.219 cells (Dox-inducible SLK carrying rKSHV.219) are latently infected with a recombinant rKSHV.219 virus and contain a doxycycline (Dox)-inducible RTA (Myoung and Ganem, 2011). The rKSHV.219 virus expresses both the red fluorescent protein (RFP) under the control of KSHV lytic PAN promoter and the green fluorescent protein (GFP) under the control of the elongation factor 1 promoter (EF-1α) (Vieira and O’Hearn, 2004). HEK293T (Human embryonic kidney 293T) cells were purchased from American Type Culture Collection (ATCC) and cultured as recommended by the manufacturer. The hiPSCs were derived originally from wild type adult skin fibroblasts, which were induced to pluripotent stem cells in Dr. Bruce Conklin’s laboratory (Miyaoka et al., 2014) and confirmed by the staining of NANOG, one of stem cell markers expression (**Figure 1**). These cells were cultivated in mTeSR1 Basal medium supplemented with 5x Supplements (Stemcell Technologies) and 1% penicillin and streptomycin (Gibco) on 1:60 growth-factor-reduced Matrigel Matrix coated dish or plates.

### 2.2 Immunofluorescence assays

Cells were seeded in eight-well chamber slides (Nunc) prior to virus infection, then fixed with 4% PFA and stained with a first antibody recognizing NANOG (Cell Signaling) or LANA (Advanced Biotechnologies), followed by a secondary antibody conjugated to Texas Red (Invitrogen) and DAPI. Fluorescence signal was measured using the Olympus IX83 microscope (Olympus).

### 2.3 Flow cytometry

Flow cytometry was used for the quantitative assessment of apoptosis with the FITC-Annexin V/propidium iodide (PI) Apoptosis Detection Kit I (BD Pharmingen) on a FACS Calibur 4-color flow cytometer (BD Bioscience). The detection of stem cell markers expression in hiPSCs using flow cytometry has been described in our previous study (Qin et al., 2011).

### 2.4 RT-qPCR

Total RNA was isolated using the RNeasy Mini kit (Qiagen), and cDNA was synthesized using a SuperScript III First-Strand Synthesis SuperMix Kit (Invitrogen). Primers used for amplification of target genes were listed in **Supplementary Table S1**. Amplification was carried out using an iCycler IQ Real-Time PCR Detection System, and cycle threshold (Ct) values were tabulated in duplicate for each gene of interest in each experiment. “No template” (water) controls were used to ensure minimal background contamination. Using mean Ct values tabulated for each gene, and paired Ct values for *β-actin* or GAPDH as a loading control, fold changes for experimental groups relative to assigned controls were calculated using automated iQ5 2.0 software (Bio-rad).

### 2.5 RNA-Sequencing and enrichment analysis

RNA-Sequencing of triplicate samples was performed by BGI Americas Corporation using their unique DNBSEQ sequencing technology. The completed RNA-Sequencing data was submitted to NCBI Sequence Read Archive (SRA# PRJNA1186964). Raw sequencing reads were analyzed using the RSEM software (version 1.3.0; human GRCh38 genome sequence and annotation) and gene expression was quantified as previously described (Kheir et al., 2019). The EBSeq software was utilized to call differentially expressed genes that were statistically significant using a false discovery rate (FDR) less than 0.05. Differentially expressed genes between cells with or without virus infection were used as input for the GO_enrichment and KEGG pathways analyses.

### 2.6 RNA interference assays

For RNA interference (RNAi) assays, FZD10 On-Target plus SMART pool small interfering RNA (siRNA; Dharmacon) or negative control siRNA, were delivered using the DharmaFECT Transfection Reagent as recommended by the manufacturer.

### 2.7 Statistical analysis

Significance for differences between experimental and control groups (with no fewer than 3 experiments per group performed) was determined using the two-tailed Student’s t-test (Excel 2016), and p values < 0.05 or < 0.01 were considered significant or highly significant, respectively.

## 3 Results

### 3.1 The hiPSCs are permissive to KSHV infection

**Figure 2:**
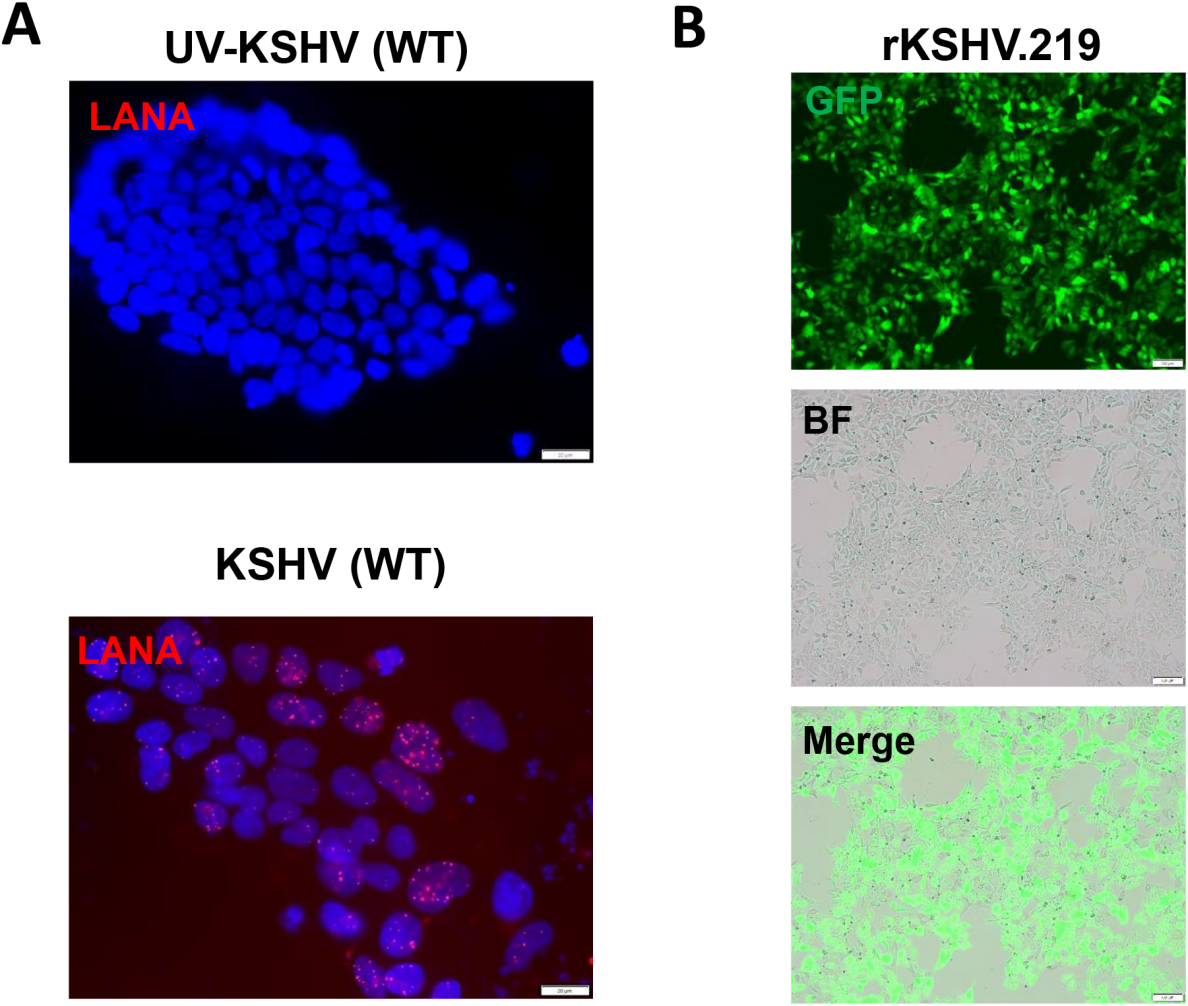
KSHV *de novo* infection of hiPSCs. **(A, B)** The hiPSCs were infected by either wild type (WT) KSHV or rKSHV.219 (MOI~3), respectively, the images were acquired by using fluorescence microscope (LANA staining for WT virus) at 72 h p.i. Scale bar: 100 μm. The UV-inactivated virus was used as a negative control.

**Figure 3:**
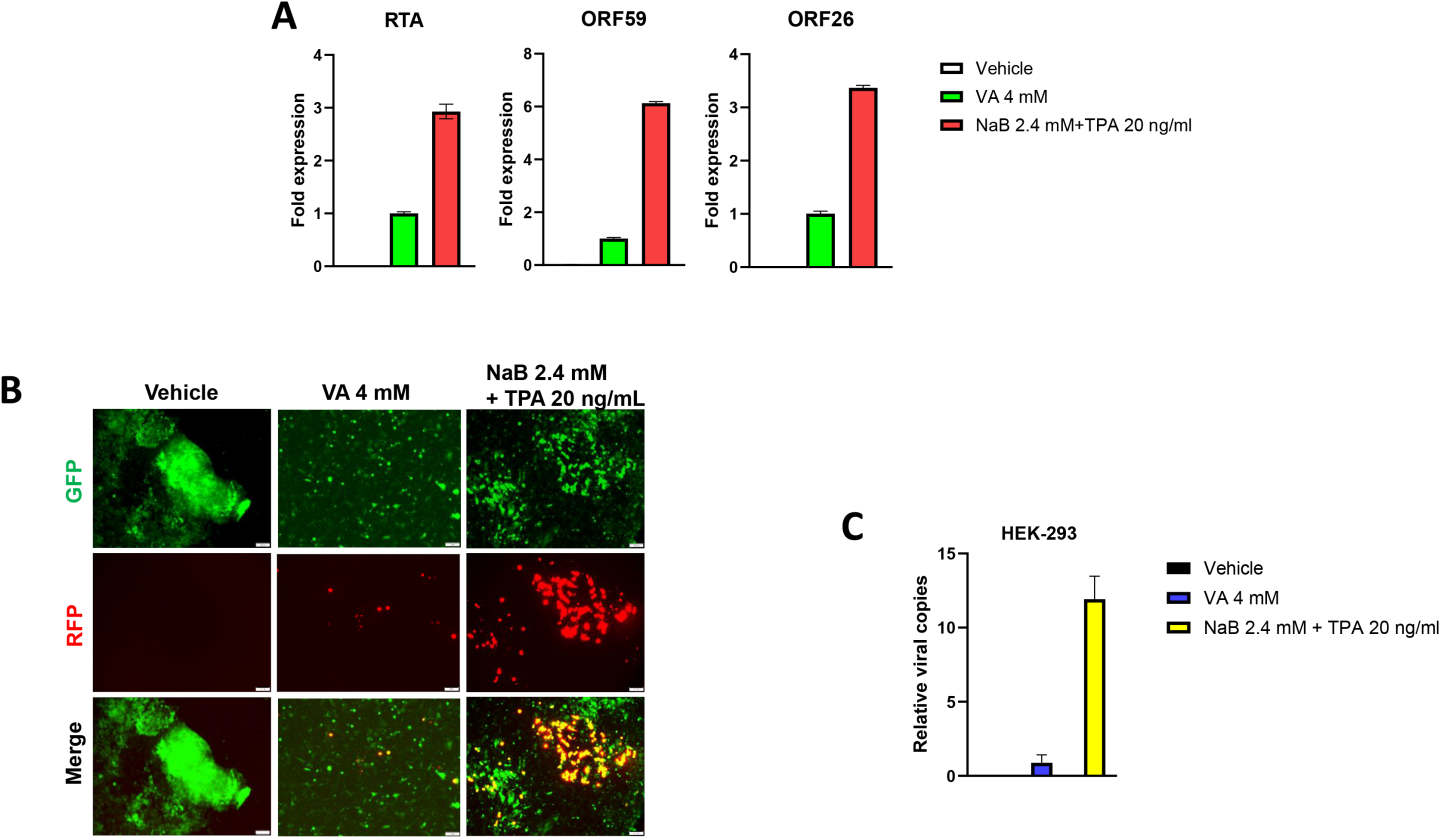
Successful induction of KSHV lytic reactivation and virions production from latently infected hiPSCs. **(A, B)** The hiPSCs were first infected by rKSHV.219 (MOI~3) for 72 h, then treated by different chemical inducers for another 72 h. The gene expression was detected and compared by using RT-qPCR (vs the VA 4 mM group), and the images were acquired by using fluorescence microscope. **(C)** The viral supernatants from **(A)** were collected to infect naïve HEK293T cells, and intracellular viral copies were detected and compared by using qPCR at 48 h p.i. The Error bars represent S.D. for 3 independent experiments. VA, valproic acid; NaB, sodium butyrate; TPA, 12-*O*-tetradecanoyl-phorbol-13-acetate.

We first tested whether hiPSCs cells (**Figure 1**) are permissive to KSHV infection. As shown in **Figure 2**, the immunofluorescence results showed that either wild type KSHV or rKSHV.219 successfully established latent infection in hiPSCs (the infection rate > 80%). We next tested whether rKSHV.219 infected cells could be induced to lytic reactivation by different chemical inducers such valproic acid (VA), sodium butyrate (NaB) and 12-*O*-tetradecanoyl-phorbol-13-acetate (TPA). Our RT-qPCR and immunofluorescence assays results showed NaB plus TPA treatment having the best effects on lytic reactivation when compared to VA alone treatment (**Figures 3A, B**). When we collected the viral supernatants to infect naïve HEK293T cells, we found that NaB plus TPA induction produced the most infectious virions through detection of intracellular viral copies by using qPCR (**Figure 3C**).

**Figure 4:**
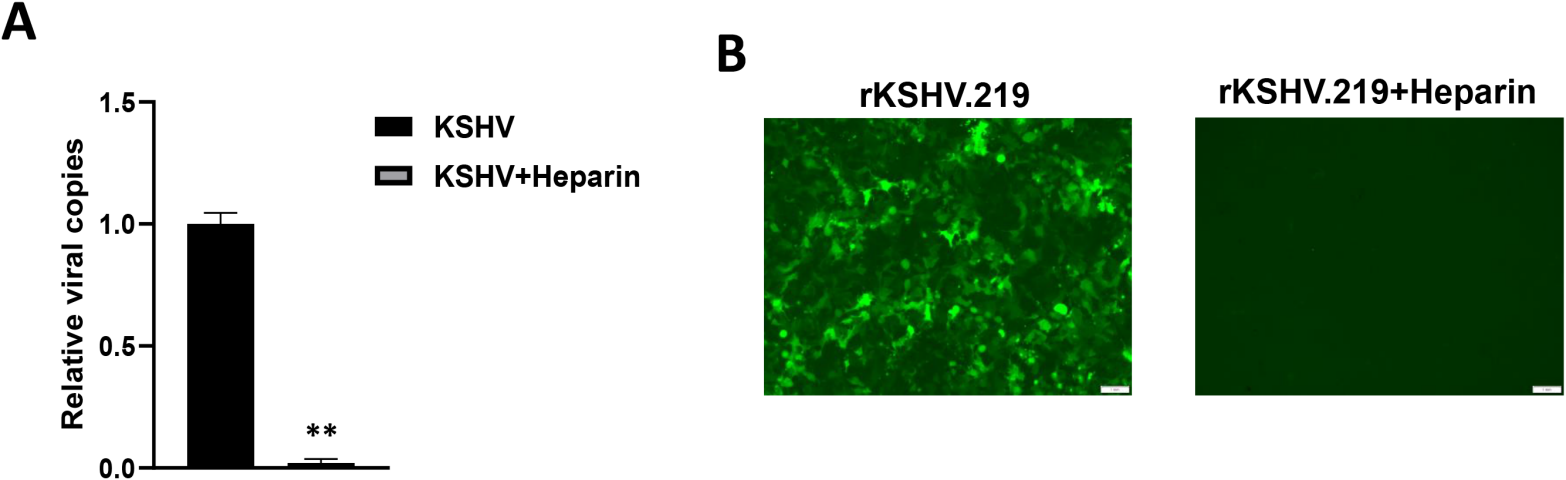
Heparan sulfate is the major cellular receptor responsible for KSHV entry into hiPSCs. **(A)** The rKSHV.219 virions were first treated with 0.5 mg/mL heparin (the competitor for heparan sulfate) for 1 h at 4°C, then the cells were infected with purified virions (MOI~3) for 2 h at 37°C. After that, cells were trypsinized and washed to remove extracellular virions. Total cellular DNA was prepared and the internalized viral copies were measured by qPCR. Error bars represent S.D. for 3 independent experiments, **p<0.01. **(B)** The images were acquired by using fluorescence microscope at 48 h p.i.

Heparan sulfate (HS) represents the major cellular receptor for KSHV entry into host cells (Birkmann et al., 2001), although it remains unclear whether HS is responsible for KSHV entry into hiPSCs. Here we found that pretreatment of rKSHV.219 virions by Heparin, the competitor of HS, almost completely blocked viral entry into hiPSCs by using qPCR and immunofluorescence assays, respectively (**Figure 4**). We observed similar results when we used wild type KSHV virions (**Supplementary Figure S1**), although other alternative receptors or pathways are probably existed for KSHV entry into hiPSCs.

### 3.2 KSHV infection arrests the growth of hiPSCs through inducing cell apoptosis

**Figure 5:**
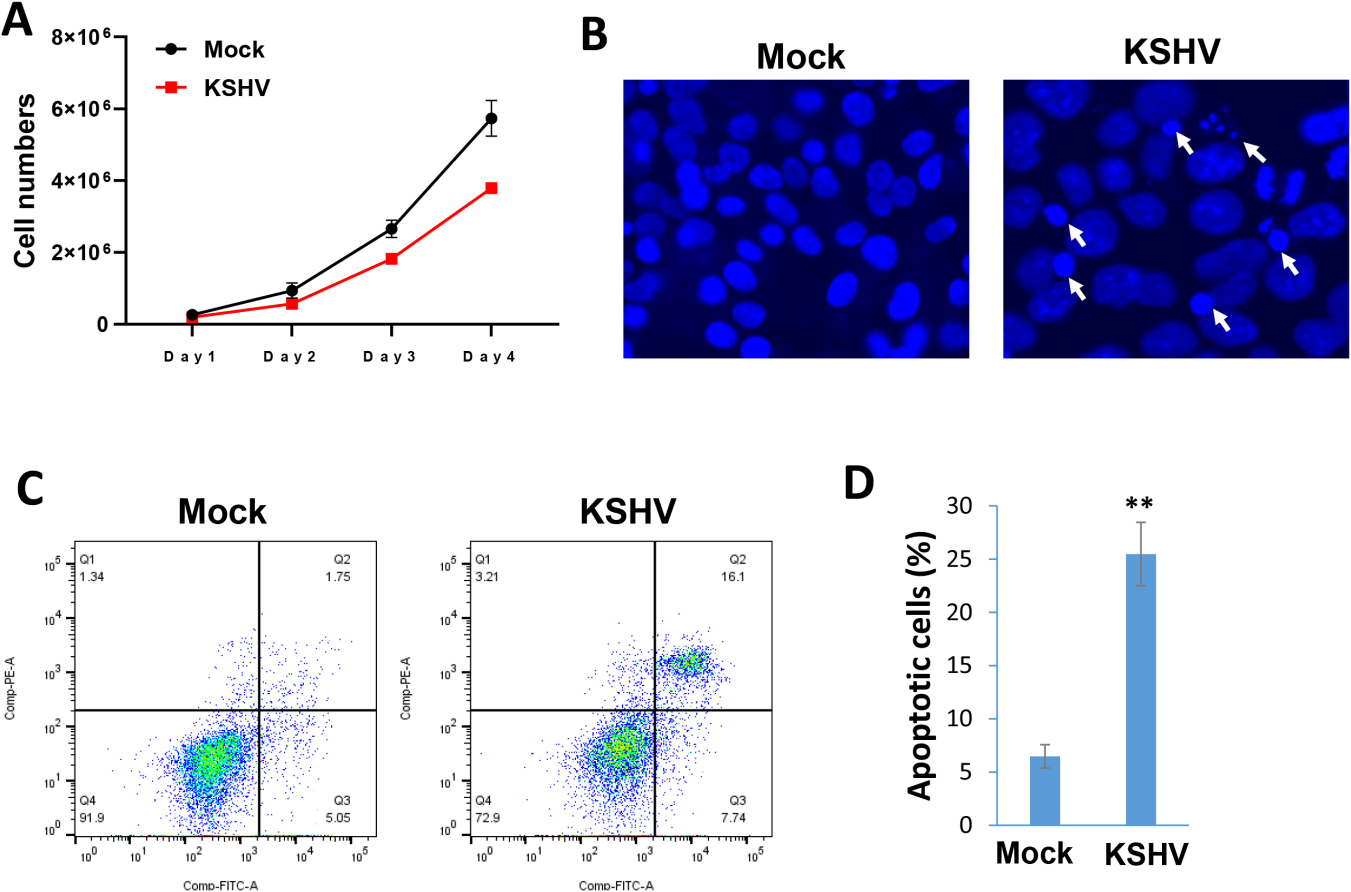
KSHV infection arrests the growth of hiPSCs through inducing cell apoptosis. **(A)** The hiPSCs-CMs were infected by KSHV, then the live cell numbers were calculated at indicated time points using Countess 3 Automated Cell Counter (Thermo Fisher). **(B)** Cells were stained by DAPI, then the images were acquired by using fluorescence microscope at 72 h p.i. The visualization of nuclear condensation or fragmentation was indicated by arrows. **(C, D)** Cell apoptosis was measured by Annexin V-PI staining and flow cytometry analysis. Error bars represent S.D. for 3 independent experiments, **p<0.01.

**Figure 6:**
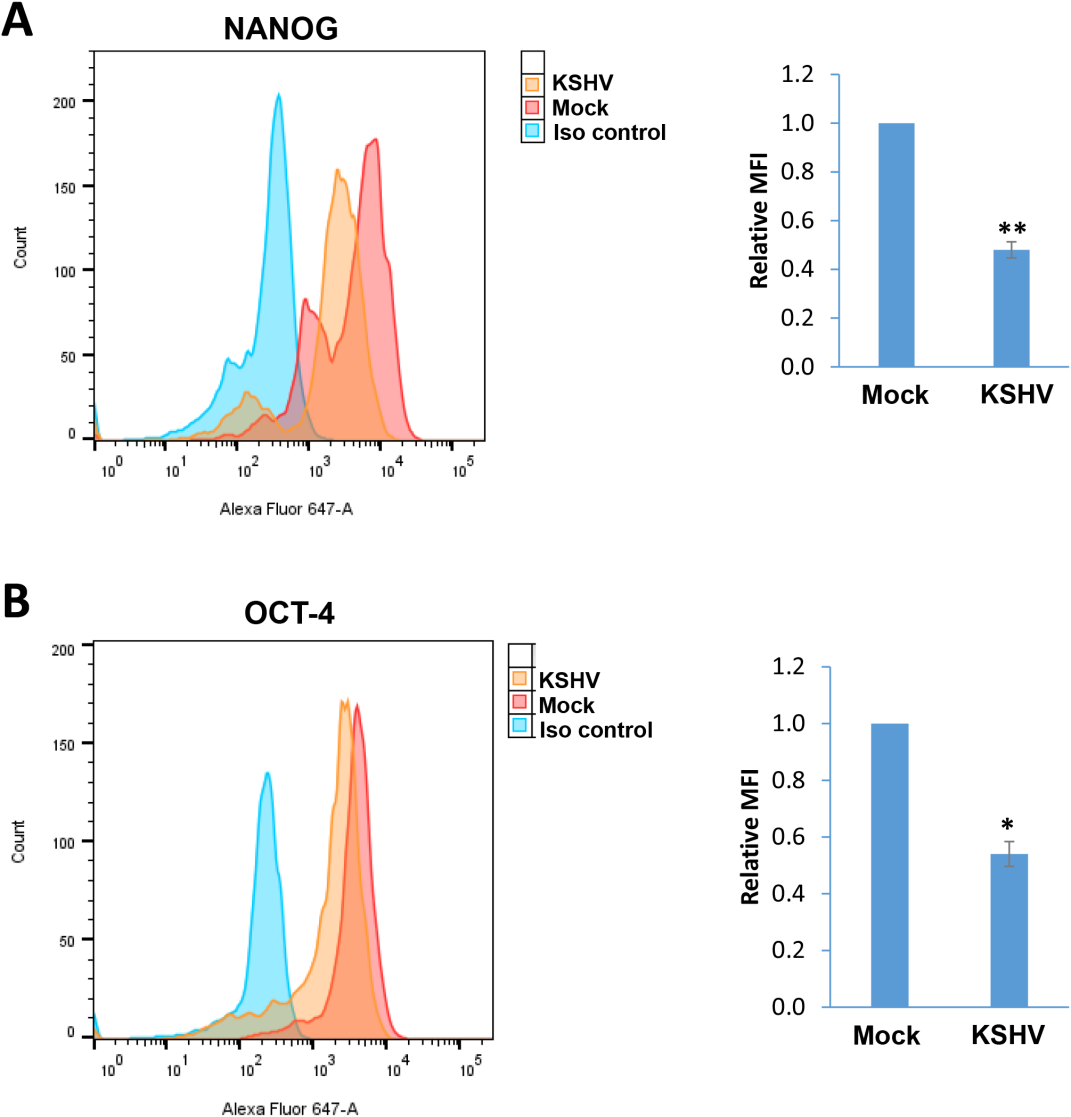
The impacts on stem cell markers expression by KSHV infection of hiPSCs. **(A, B)** Flow cytometry analyses were used to quantify NANGO and OCT-4 expression in mock- or KSHV-infected hiPSCs. Mean fluorescence intensity (MFI), reflecting the expression of each protein for the same number of cells in each condition was calculated using FlowJo software. Error bars represent S.D. for 3 independent experiments, *p<0.05, **p<0.01.

In a time-course assay, we found that KSHV infection caused growth arrest of hiPSCs when compared to non-infected cells (**Figure 5A**). Using the DAPI staining, we observed obvious nuclear condensation or fragmentation only in KSHV-infected cells (**Figure 5B**). Our flow cytometry analysis further confirmed that KSHV infection caused increased cell apoptosis in hiPSCs (**Figures 5C, D**). We also examined the impacts of KSHV infection on the expression of stem cell markers from hiPSCs by flow cytometry analysis. Our results showed that the expression of NANOG and OCT-4 was significantly downregulated (~50% of reduction) in KSHV-infected hiPSCs (**Figure 6**). As a control, NANOG and OCT-4 are almost not expressed on BCBL-1 cells (**Supplementary Figure S2**).

### 3.3 Transcriptomic analysis of gene profiling changed in KSHV-infected hiPSCs cells

**Figure 7:**
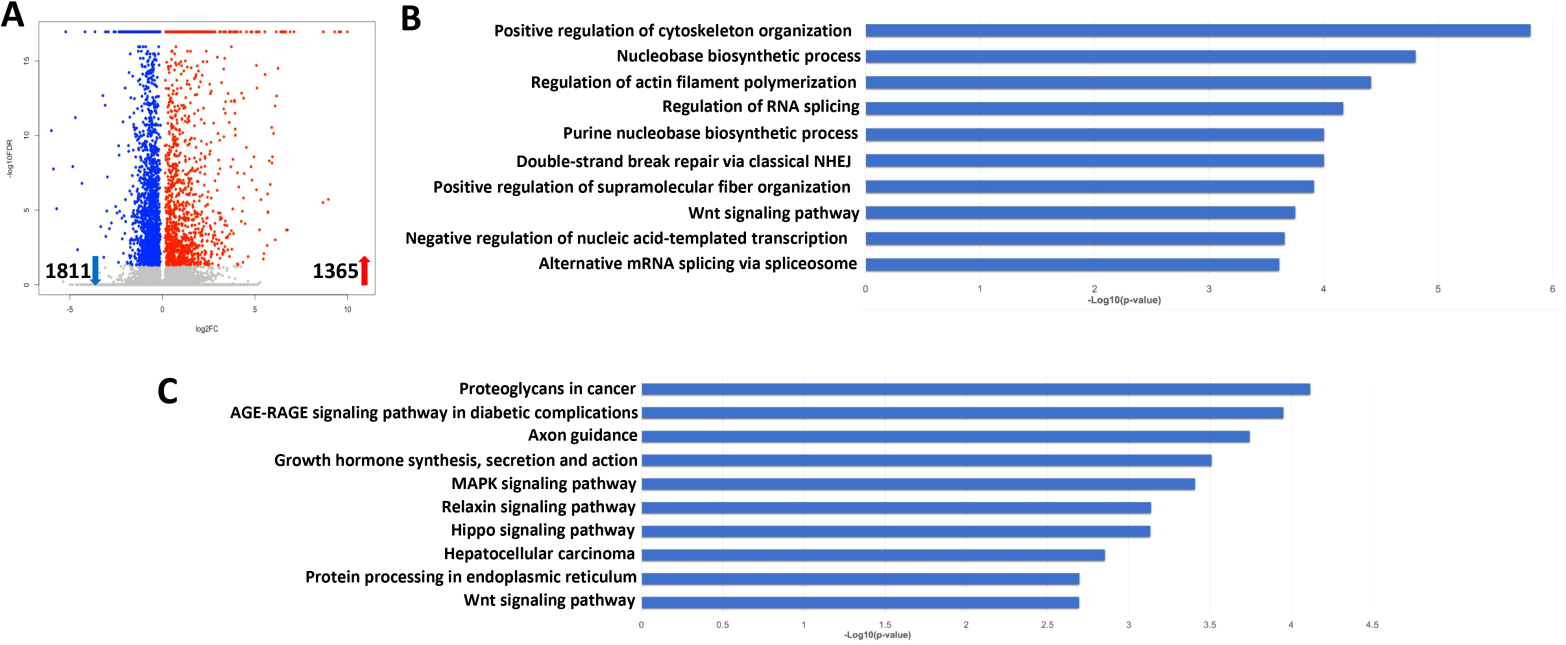
Transcriptome analysis of KSHV latently infected hiPSCs. **(A)** RNA-Sequencing was used to investigate changes in the transcriptome from rKSHV.219 infected cells (72 h p.i.) when compared to mock cells. The significantly altered genes (FDR<0.05) were shown in the Volcano plot panel. **(B, C)** The GO_enrichment (Biological process module) and KEGG pathway analysis of the significantly changed genes in rKSHV.219 infected cells.

To determine the global cellular changes caused by KSHV infection, we compared the gene profiles of mock- and KSHV-infected hiPSCs, using RNA-Sequencing analyses. The volcano plots showed the scattering of genes which were significantly upregulated (1365 transcripts) or downregulated (1811 transcripts) in KSHV-infected hiPSCs (**Figure 7A**). The top 20 significantly upregulated or downregulated genes in KSHV-infected hiPSCs were listed in **Table 1**. The GO_enrichment (Biological process module) analysis of these significantly changed genes identified several major functional categories potentially involved, such as cytoskeleton organization and functions, regulation of RNA splicing, DNA repair, nucleobase biosynthetic process, etc (**Figure 7B**). The KEGG analysis indicated that KSHV infection affected pathways important for MAPK, Relaxin, Hippo and Wnt signaling in hiPSCs (**Figure 7C**). Among them, the Hippo and Wnt signaling pathways play important roles in stem cells functions and development (Zhao et al., 2024; Yu et al., 2024). The bioinformatics analysis predicted that the changed candidates (42 in Hippo pathway and 41 in Wnt pathway, respectively) showed a close protein-protein interaction network and potential functional association (**Supplementary Figure S3**).

**Table 1:** The top 20 candidate genes upregulated or downregulated within KSHV latently infected hiPSCs.

Gene	Fold change	Description
CXCR5	415.0	C-X-C motif chemokine receptor 5
ZACN	407.7	zinc activated ion channel
FZD10	93.5	frizzled class receptor 10
FEZF2	71.1	FEZ family zinc finger 2
AJAP1	68.0	adherens junctions associated protein 1
ELAVL4	64.0	ELAV like RNA binding protein 4
DPPA3	54.6	developmental pluripotency associated 3
HCG25	50.6	HLA complex group 25
CHP2	46.4	calcineurin like EF-hand protein 2
SHISA6	40.1	shisa family member 6
NR2F2	35.2	nuclear receptor subfamily 2 group F member 2
MT1F	28.7	metallothionein 1F
S100A6	27.6	S100 calcium binding protein A6
ADCY8	23.3	adenylate cyclase 8
SIX3	23.1	SIX homeobox 3
RGN	19.3	regucalcin
MMRN1	19.2	multimerin 1
RARRES3	16.6	phospholipase A and acyltransferase 4
CHODL	15.9	chondrolectin
DLK1	15.8	delta like non-canonical Notch ligand 1
AKR1B10	0.02	aldo-keto reductase family 1 member B10
WNT8A	0.03	Wnt family member 8A
UQCR11	0.03	ubiquinol-cytochrome c reductase, complex III subunit XI
KRT81	0.04	keratin 81
CYP24A1	0.05	cytochrome P450 family 24 subfamily A member 1
CES1	0.05	carboxylesterase 1
GBX2	0.08	gastrulation brain homeobox 2
CALCRL	0.12	calcitonin receptor like receptor
ERAP2	0.16	endoplasmic reticulum aminopeptidase 2
APOLD1	0.20	apolipoprotein L domain containing 1
CCND1	0.20	cyclin D1
CXCL1	0.21	C-X-C motif chemokine ligand 1
TNMD	0.23	tenomodulin
HHAT	0.23	hedgehog acyltransferase
ALDH3A1	0.25	aldehyde dehydrogenase 3 family member A1
SLC44A5	0.26	solute carrier family 44 member 5
FBLN7	0.26	fibulin 7
FUT5	0.27	fucosyltransferase 5
PDGFA	0.27	platelet derived growth factor subunit A
EFNB1	0.29	ephrin B1

**Figure 8:**
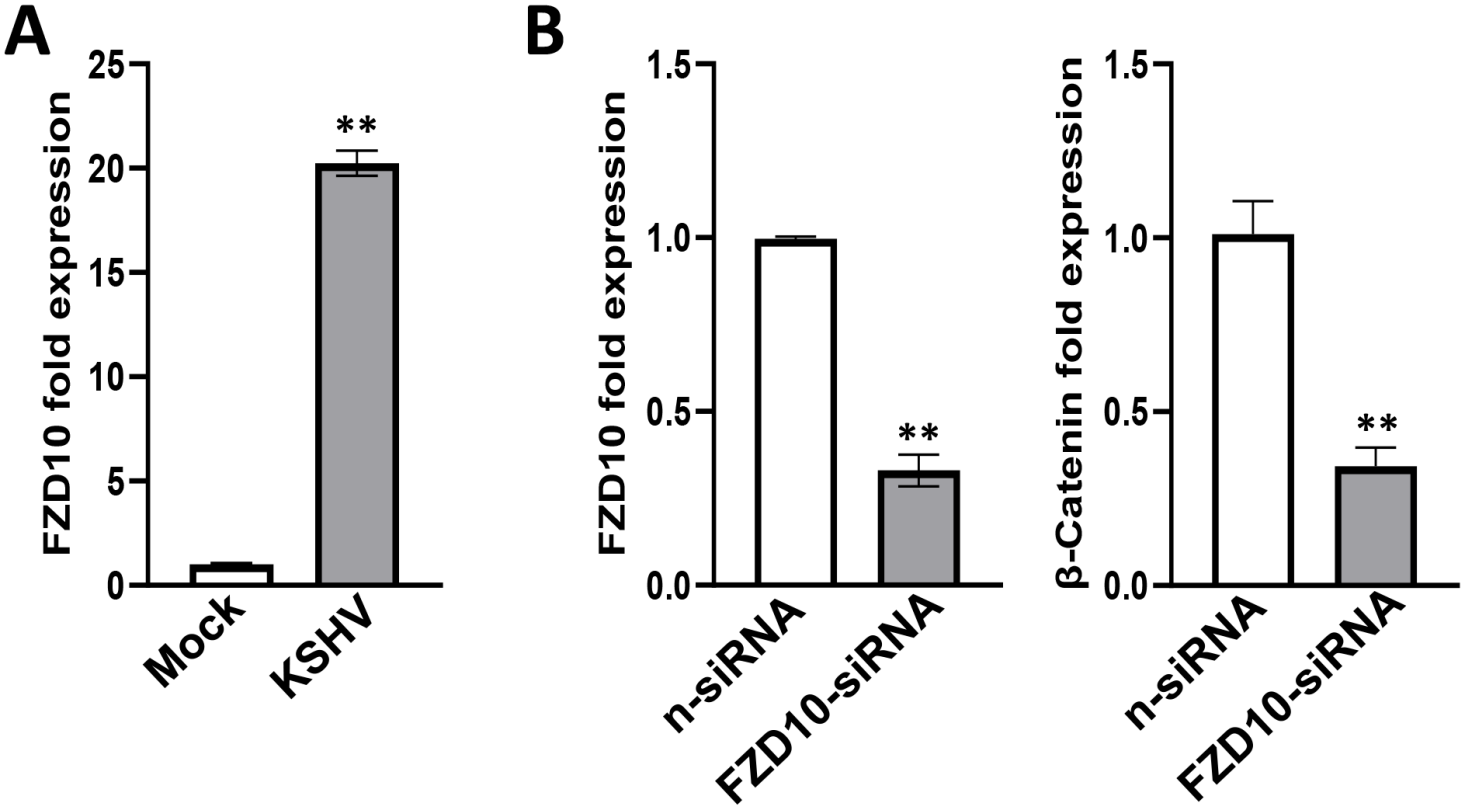
Functional validation of FZD10 candidate gene in KSHV-infected hiPSCs. **(A)** The hiPSCs were first infected by rKSHV.219 (MOI~3) for 72 h, then gene expression was detected and compared by using RT-qPCR. **(B)** The hiPSCs were first transfected with FZD10-siRNA or non-target control siRNA (n-siRNA) for 48 h, then infected by rKSHV.219. Gene expression was detected and compared by using RT-qPCR. Error bars represent S.D. for 3 independent experiments, **p<0.01.

One of upregulated candidate genes identified from RNA-Sequencing analyses, frizzled class receptor 10 (FZD10), is a member of the frizzled gene family. Members of this family encode 7-transmembrane domain proteins that are receptors for the Wnt signaling protein ligands. Most frizzled receptors including FZD10 are coupled to the β-Catenin canonical signaling pathway (Katoh and Katoh, 2017). Our RT-qPCR results further confirmed that FZD10 transcriptional level was dramatically increased from KSHV-infected hiPSCs (**Figure 8A**). We then silenced FZD10 with RNAi, and found that it could significantly reduce the level of β-Catenin transcription (**Figure 8B**).

**Figure 9:**
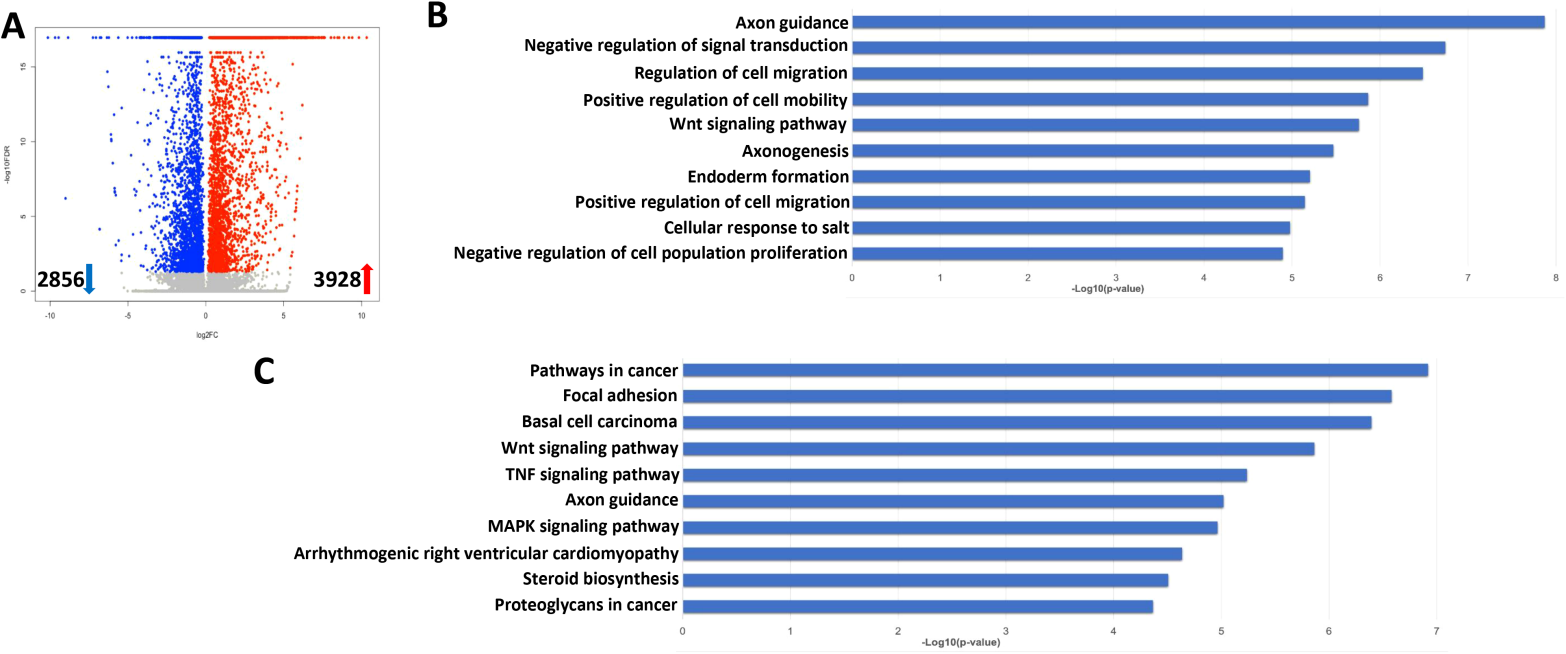
Transcriptome analysis of lytically induced KSHV infected hiPSCs. **(A)** RNA-Sequencing was used to investigate changes in the transcriptome from NaB/TPA induced rKSHV.219 infected cells (72 h) when compared to non-induced cells. The significantly altered genes (FDR<0.05) were shown in the Volcano plot panel. **(B, C)** The GO_enrichment (Biological process module) and KEGG pathway analysis of the significantly changed genes from infected cells.

We further explored the gene profiles changed in lytically induced KSHV-infected hiPSCs by NaB plus TPA. The volcano plots showed the scattering of genes which were significantly upregulated (3928 transcripts) or downregulated (2856 transcripts) in lytically induced cells (**Figure 9A**). The top 20 significantly upregulated or downregulated genes in lytically induced cells were listed in **Table 2**. The GO_enrichment analysis of these significantly changed genes identified several major functional categories, including regulation of cell migration/mobility, axonogenesis, regulation of cell proliferation, etc (**Figure 9B**). The KEGG analysis indicated that induction of lytic reactivation affected pathways important for focal adhesion, steroid biosynthesis, cellular signaling such as Wnt, TNF and MAPK (**Figure 9C**).

**Table 2:** The top 20 candidate genes upregulated or downregulated within lytically induced KSHV-infected hiPSCs.

Gene	Fold change	Description
SELE	1284.6	selectin E
TNFRSF6B	909.7	TNF receptor superfamily member 6b
CCL20	674.3	C-C motif chemokine ligand 20
TNF	468.5	tumor necrosis factor
RAB7B	378.4	RAB7B, member RAS oncogene family
TMPRSS11E	350.8	transmembrane serine protease 11E
MAGEB2	263.4	MAGE family member B2
CST5	260.2	cystatin D
CST11	197.6	cystatin 11
TNFRSF9	197.1	TNF receptor superfamily member 9
SPATA31A6	191.8	SPATA31 subfamily A member 6
SPATA31A7	189.2	SPATA31 subfamily A member 7
PAEP	183.5	progestagen associated endometrial protein
BRDT	182.7	bromodomain testis associated
ARHGAP40	173.4	Rho GTPase activating protein 40
CST2	172.3	cystatin SA
ADAM18	154.2	ADAM metallopeptidase domain 18
EREG	147.7	epiregulin
SPATA31A3	145.5	SPATA31 subfamily A member 3
KCNH3	134.2	potassium voltage-gated channel subfamily H member 3
GPS2	0.01	G protein pathway suppressor 2
HES3	0.01	hes family bHLH transcription factor 3
CD1C	0.02	CD1c molecule
GADD45G	0.02	growth arrest and DNA damage inducible gamma
RARRES3	0.04	phospholipase A and acyltransferase 4
CYP2A13	0.04	cytochrome P450 family 2 subfamily A member 13
PYDC1	0.05	pyrin domain containing 1
TDGF1	0.05	cripto, EGF-CFC family member
LEFTY1	0.05	left-right determination factor 1
HMX2	0.05	H6 family homeobox 2
PCSK9	0.05	proprotein convertase subtilisin/kexin type 9
ACTN3	0.06	actinin alpha 3
MMP24	0.06	matrix metallopeptidase 24
METTL7A	0.06	thiol methyltransferase 1A
LPL	0.06	lipoprotein lipase
SPTSSB	0.06	serine palmitoyltransferase small subunit B
FOXH1	0.06	forkhead box H1
FGFBP3	0.06	fibroblast growth factor binding protein 3
INMT	0.07	indolethylamine N-methyltransferase
TDGF1P3	0.08	cripto, EGF-CFC family member 3

## 4 Discussion

As mentioned above, the new technology allows us to generate hiPSCs without human embryo, which can overcome the issues of histoincompatibility and ethical problems associated with the use human embryonic stem cells (Senju et al., 2011). Since KSHV can infect a broad tropism of human primary cells, some of them are not easily isolated from the donors or no commercial cell lines available. Thus, the hiPSCs may represent a powerful tool to study KSHV infection and pathogenesis in different types of differentiated cells. For instance, recent data indicate that KSHV infection is potentially associated with some heart diseases such as dilated cardiomyopathy (DCM) (Zhao et al., 2023; Huang et al., 2024; Hsueh et al., 2006). Zhao and colleague reported that increased KSHV seropositivity and quantitative titers were found in the patients with DCM compared with the non-DCM group. The risk of the individual end point of death from cardiovascular causes or heart transplantation was increased among DCM patients with the KSHV DNA seropositivity during follow-up (Zhao et al., 2023). As we know, primary isolated cardiomyocytes are very difficult to culture *in vitro*, so the hiPSCs induced cardiomyocytes will be a better option for such research.

In the current study, we found that the hiPSCs were permissive to KSHV infection including being completely induced to lytic reactivation and releasing infectious virions. Also, we identified heparan sulfate as the major cellular receptor for KSHV entry into hiPSCs. Interestingly, one recent study reported Neuropilin 1 as an entry receptor for KSHV infection of MSCs through TGFBR1/2-mediated micropinocytosis (Lu et al., 2023). In that study, the authors used different types of oral MSCs, including periodontal ligament stem cells (PDLSC), dental pulp stem cells (DPSC), and gingiva/mucosa-derived mesenchymal stem cells (GMSC). These data together with ours suggest that KSHV may use different receptors to entry into stem cells with varied origins.

Using the hiPSCs, we can also study the impacts of KSHV infection on these stem cells differentiation. Previous study found that KSHV infection of oral MSCs promoted multilineage differentiation and mesenchymal-to-endothelial transition (MEndT) (Li et al., 2018). Here, our transcriptomic analysis indicated that KSHV infection affected several cellular signaling pathways important for stem cells differentiation, such as Hippo and Wnt pathways. Previous study reported that KSHV-encoded G-protein coupled receptor (vGPCR) protein was able to inhibit the Hippo pathway kinases Lats1/2, promoting the activation of YAP/TAZ, cell proliferation and tumorigenesis (Liu et al., 2015). Interestingly, the vGPCR protein was found to activate the canonical Wnt/β-catenin signaling activities, which was dependent on the PI3K/Akt pathway (Angelova et al., 2014). Another viral protein, latency-associated nuclear antigen (LANA), was also found to regulate the activities of Wnt signaling pathway (Kusano and Eizuru, 2010). However, the molecular mechanisms of KSHV manipulating these signaling pathways to modulate hiPSCs differentiation and other functions still require further investigation. Notably, there is a close relationship between the Hippo pathway and the canonical Wnt pathway, and their crosstalk may play a role in some human diseases (Sileo et al., 2022).

## Data Availability

The datasets presented in this study can be found in online repositories. The names of the repository/repositories and accession number(s) can be found below: https://www.ncbi.nlm.nih.gov/, SRA# PRJNA1186964.
