# Supplementary material for: Infection of human induced pluripotent stem cells by an oncogenic herpesvirus
Source: Front Cell Infect Microbiol. 2025 Mar 13;15:1563440. doi: 10.3389/fcimb.2025.1563440 (PMC11966036; doi:10.3389/fcimb.2025.1563440)
Supplement: Supplementary file 1 [file DataSheet1.pdf]

Supplementary Table 1. Primer sequences for qPCR and RT-qPCR

	Gene	Forward primers	Reverse primers
qPCR	LANA	5'-TCCCTCTACACTAAACCCAATA-3'	5'-TTGCTAATCTCGTTGTCCC-3'
	GAPDH	5'-GCTCCCTCTTTCTTTGCAGCAAT-3'	5'-TACCATGAGTCCTTCCACGATAC-3'
RT-qPCR	RTA	5’-CACAAAAATGGCGCAAGATGA-3’	5’-TGGTAGAGTTGGGCCTTCAGTT-3’
	ORF59	5’-CGAGTCTTCGCAAAAGGTTC-3’	5’-AAGGGACCAACTGGTGTGAG-3’
	ORF26	5’-GCTCGAATCCAACGGATTTG-3’	5’-AATAGCGTGCCCCAGTTGC-3’
	FZD10	5’-ATCTGGGCGGTGCTGTGCT-3’	5’-CGAAGAGGCGGATGAGGT-3’
	β-catenin	5’-TGCCAAGTGGGTGGTATA-3’	5’-GGGATGGTGGGTGTAAGA-3’
	β-actin	5’-ATCGTGCGTGACATTAAGGAG-3’	5’-GGAAGGAAGGCTGGAAGAGT-3’

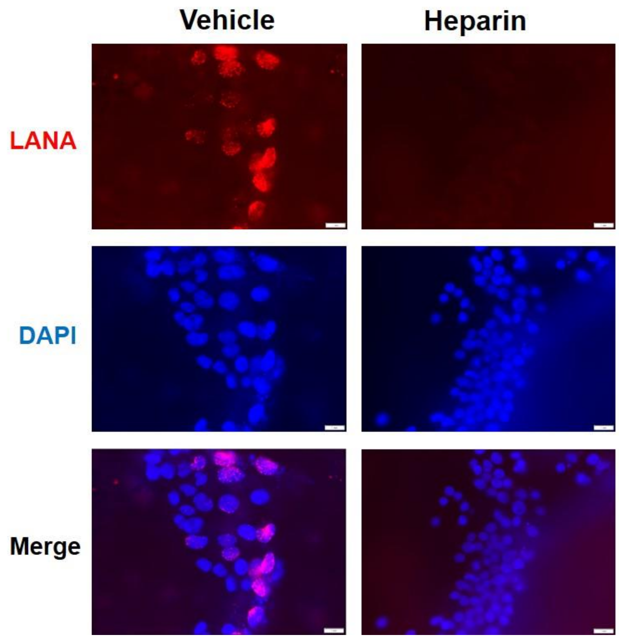

Figure S1. Heparan sulfate is the major cellular receptor responsible for KSHV entry into hiPSCs. The wild type KSHV virions were first treated with 0.5 mg/mL heparin (the competitor for heparan sulfate) for 1 h at 4°C, then the cells were infected with purified virions (MOI~3) for 2 h at 37°C. The images were acquired by using fluorescence microscope (LANA staining) at 48 h p.i.

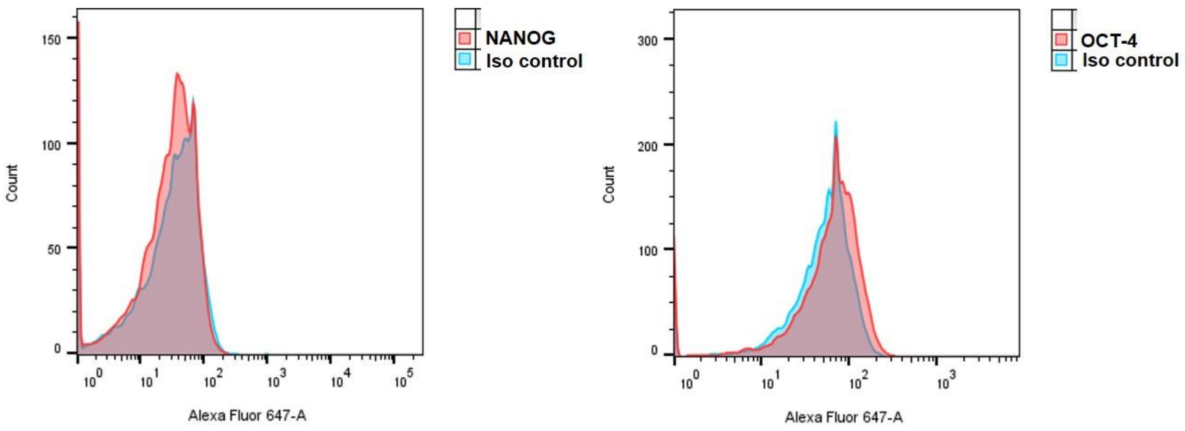

Figure S2. Detection of stem cell markers expression on KSHV+ tumor cells. Flow cytometry was used to detect NANGO and OCT-4 expression on BCBL-1 cells.

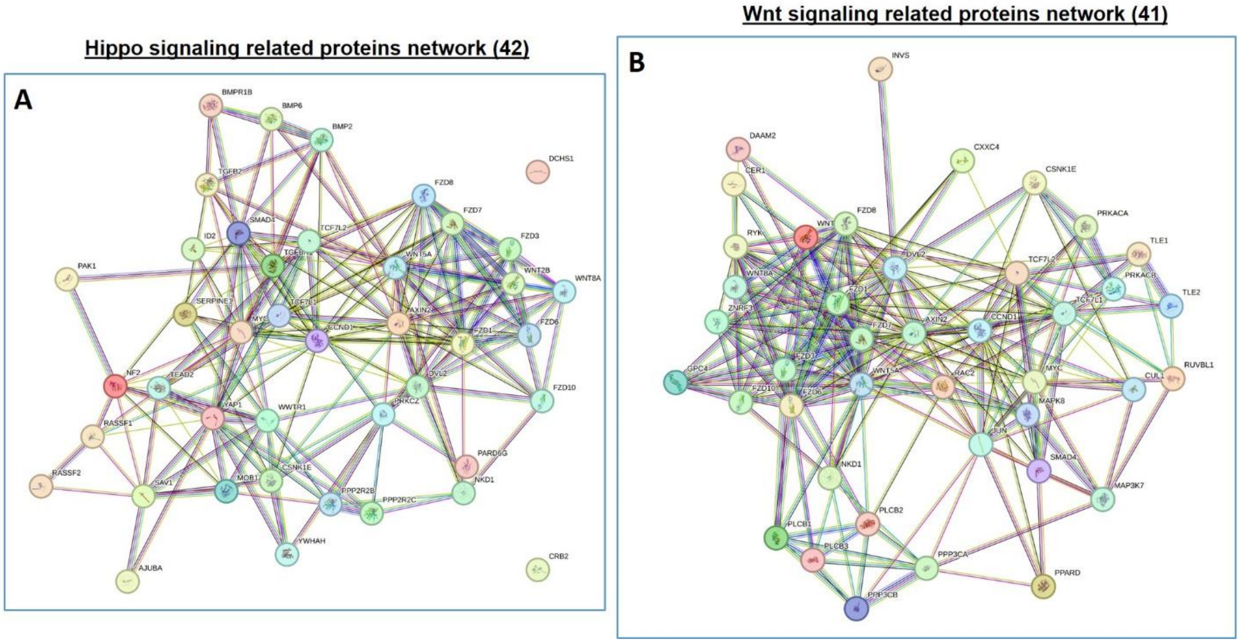

Figure S3. Protein-protein interaction network in Hippo and Wnt signaling pathways affected by KSHV in hiPSCs. (**A-B**) Protein-protein interaction analysis was performed at https://strin g-db.org/. The representation of action types and node colours has been listed on the website.
